# Correction: Deciphering the molecular origin of the 19.3 eV electronic excitation energy of H_3_^+^

**DOI:** 10.1039/d6sc90041k

**Published:** 2026-02-17

**Authors:** Josene M. Toldo, Jakob K. Staab, Eduard Matito, Cina Foroutan-Nejad, Henrik Ottosson

**Affiliations:** a Department of Chemistry – Ångström, Uppsala University 751 20 Uppsala Sweden henrik.ottosson@kemi.uu.se; b Université Claude Bernard Lyon 1, ENS de Lyon, CNRS, Laboratoire de Chimie, UMR 5182 69342 Lyon Cedex 07 France; c Department of Chemistry, The University of Manchester Oxford Road Manchester UK; d Donostia International Physics Center (DIPC) 20018 Donostia Euskadi Spain; e Ikerbasque, Basque Foundation for Science 48009 Bilbao Euskadi Spain; f Institute of Organic Chemistry, Polish Academy of Sciences Warsaw Poland

## Abstract

Correction for ‘Deciphering the molecular origin of the 19.3 eV electronic excitation energy of H_3_^+^’ by Josene M. Toldo *et al.*, *Chem. Sci.*, 2026, https://doi.org/10.1039/d5sc09067a.

The authors regret that panel A of Fig. 4 in the original article was not complete as its right part, with results for the excited state labelled 1^1^B_2_, was accidently omitted. The missing part, with the topological analysis of the electron density, the 2D Laplacian of the electron density, and the natural orbitals of the 1^1^B_2_ state, is contained in the new Fig. 4 shown as follows.

**Fig. 4:**
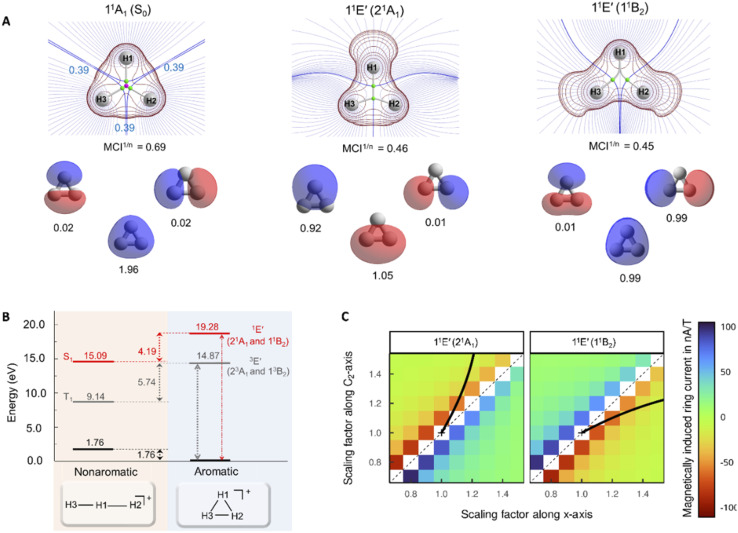
(A) Topological analysis of the electron density, 2D Laplacian of the electron density (in red), and natural orbitals (with populations) for the S_0_ and 1^1^E′ states, the latter labelled as 2^1^A_1_ and 1^1^B_2_ in *C*_2v_ symmetry. The rays of the basins drawn in blue and density gradient lines in purple. MCI^1/*n*^ values (computed using Becke-rho’s partition)^56^ are given below the Laplacian plots of the electron density. (B) Vertical excitation energies and relative energies of H_3_^+^ at, respectively, *D*_∞h_ and *D*_3h_ symmetries. (C) Magnetically induced ring currents for the 2^1^A_1_ and 1^1^B_2_ states which stem from the 1^1^E′ states upon geometric distortions to *C*_2v_ symmetric structures. The scaling factors reflect how large this distortion was (the value 1.0 represents the H–H bond lengths of the S_0_ equilibrium geometry). The *C*_2_-axis indicates distortions in the direction of forming an acute isosceles triangle (moving H1 atom) and the *x*-axis distortions along an obtuse isosceles triangle formation (increasing the separation between H2 and H3).

The Royal Society of Chemistry apologises for these errors and any consequent inconvenience to authors and readers.

